# Enhancing anti-tumour innate immunity by targeting the DNA damage response and pattern recognition receptors in combination with radiotherapy

**DOI:** 10.3389/fonc.2022.971959

**Published:** 2022-08-29

**Authors:** Charleen M. L. Chan Wah Hak, Antonio Rullan, Emmanuel C. Patin, Malin Pedersen, Alan A. Melcher, Kevin J. Harrington

**Affiliations:** ^1^ Translational Immunotherapy Team, The Institute of Cancer Research, London, United Kingdom; ^2^ Targeted Therapy Team, The Institute of Cancer Research, London, United Kingdom

**Keywords:** DNA damage, innate immunity, radiotherapy, immunotherapy, combination therapy, cancer therapy

## Abstract

Radiotherapy is one of the most effective and frequently used treatments for a wide range of cancers. In addition to its direct anti-cancer cytotoxic effects, ionising radiation can augment the anti-tumour immune response by triggering pro-inflammatory signals, DNA damage-induced immunogenic cell death and innate immune activation. Anti-tumour innate immunity can result from recruitment and stimulation of dendritic cells (DCs) which leads to tumour-specific adaptive T-cell priming and immunostimulatory cell infiltration. Conversely, radiotherapy can also induce immunosuppressive and anti-inflammatory mediators that can confer radioresistance. Targeting the DNA damage response (DDR) concomitantly with radiotherapy is an attractive strategy for overcoming radioresistance, both by enhancing the radiosensitivity of tumour relative to normal tissues, and tipping the scales in favour of an immunostimulatory tumour microenvironment. This two-pronged approach exploits genomic instability to circumvent immune evasion, targeting both hallmarks of cancer. In this review, we describe targetable DDR proteins (PARP (poly[ADP-ribose] polymerase); ATM/ATR (ataxia–telangiectasia mutated and Rad3-related), DNA-PKcs (DNA-dependent protein kinase, catalytic subunit) and Wee1 (Wee1-like protein kinase) and their potential intersections with druggable immunomodulatory signalling pathways, including nucleic acid-sensing mechanisms (Toll-like receptors (TLR); cyclic GMP–AMP synthase (cGAS)–stimulator of interferon genes (STING) and retinoic acid-inducible gene-I (RIG-I)-like receptors), and how these might be exploited to enhance radiation therapy. We summarise current preclinical advances, recent and ongoing clinical trials and the challenges of therapeutic combinations with existing treatments such as immune checkpoint inhibitors.

## 1 Introduction

Radiotherapy continues to be one of the most effective treatments for a wide range of cancers since its discovery over a century ago. Approximately half of cancer patients receive radiotherapy at some point in their cancer treatment (1), whether in the curative or palliative settings.

Radiotherapy exploits ionising radiation to cause cell death or senescence *via* DNA damage. Broadly, necrotic or apoptotic cell death occurs depending on cell type, radiotherapy dose and fractionation schedule (2). Cancer cells that evade apoptosis and continue to divide with accumulated DNA damage can die *via* mitotic catastrophe. Also, excess autophagy can force the cell into apoptotic or necrotic cell death (3, 4). Classically, the response of tumours to conventional fractionated radiotherapy is governed by the principles of the 4 “R”s of radiobiology: *repair* of sublethal DNA damage after exposure to ionising radiation, *redistribution* of cells in the cell cycle whereby cells in the G2/M-phase are most radiosensitive and are preferentially killed in comparison to the more radioresistant late S-phase, *repopulation* of tumour cells and r*eoxygenation* of previously hypoxic tumour areas (5). A 5^th^ “R” of intrinsic *radiosensitivity* has also postulated by Steel, after observing the varying survival curves of different tumour cell lines following irradiation, which is thought to be independent of their DNA repair capacity (6). Combining agents that can target DNA damage repair pathways, as one of the 4 “R”s, with radiotherapy holds considerable potential to enhance therapeutic outcomes.

In addition to direct cell killing, radiotherapy can induce immunogenic cell death (ICD) and modulate the immune tumour microenvironment to lead to anti-tumour innate immune activation (7). Due to these immunostimulatory effects, there is increased interest in radiotherapy as a promising combinatorial agent with other immuno-oncology agents such as DNA-damage response (DDR)-targeting agents (8). This two-pronged approach exploits two hallmarks of cancer, namely genomic instability and evasion of immune surveillance (9, 10). The DDR sensing and signalling pathway are the collective mechanisms evolved by cells to combat the threat of DNA damage, namely the detection of DNA lesions, signalling of their presence and promotion of DNA repair (11). Promising DDR druggable targets include those within DNA repair pathways and cell cycle checkpoints, as well as damage-associated molecular pattern (DAMP)-sensing receptors which can amplify the DDR-induced immune response when combined with radiotherapy.

## 2 Radiotherapy and the anti-tumour immune response

Radiotherapy has both immunostimulatory and immunosuppressive effects. The difference in the ability of radiotherapy to initiate pro-immunostimulatory effects and turn immunogenically “cold” (low T-cell infiltrated) tumours “hot” (high T-cell infiltrated) may account for the enhanced response to radiotherapy of some pre-clinical models and clinical cancer histotypes.

### 2.1 Immunostimulatory effects mediated by radiotherapy

#### 2.1.1 Immunogenic cell death 

As a defence against microbial infection, the innate immune system has evolved pattern-recognition receptors (PRRs) that detect microbial pathogenic molecules known as pathogen-associated molecular patterns (PAMPs). However, these pathways do not exclusively sense foreign molecules. Immune activation can also occur in the absence of microbial infection, instead being triggered by inflammatory signals released from stressed or dying cells collectively known as damage-associated molecular patterns (DAMPs) (12). Radiotherapy-induced cellular stress and ICD can stimulate an immune response through the generation of DAMPs (13) detected by their cognate pattern recognition receptors (PRRs) (14). ICD has been defined as the chronic exposure of DAMPs in the tumour environment (TME), which can induce an innate and adaptive anti-tumour immune response in the host (15).

A characteristic DAMP induced by ICD is the secretion of adenosine triphosphate (ATP) from dying cancer cells into the extracellular space. Extracellular ATP functions as a “find-me” chemoattractant signal for the recruitment and activation of dendritic cells (DCs) (15–17). High-mobility group box-1 (HMGB1), secreted from the nucleus during ICD, binds to Toll-like receptor (TLR-4) and is critical for activating DCs and facilitating antigen processing and presentation to T cells (18). Translocation of calreticulin to the cell surface on dying cells provides an “eat-me” signal to antigen-presenting cells (APCs) and results in their phagocytosing target cells (19). In the context of cancer, ICD leads to release of tumour-associated antigens (TAA) and subsequent priming of a cancer-specific immune response. Another characteristic of ICD is the expression of heat shock proteins (HSP) HSP70 and HSP90 on dying cell membranes that drives cross-presentation of tumour-derived antigens on major histocompatibility complex class I (MHC-I) (15).

#### 2.1.2 Secretion of pro-inflammatory mediators

Radiotherapy-induced DNA damage can function as a viral mimic through the accumulation of cytosolic DNA or RNA in irradiated cells (20). Cytosolic DNA and RNA activate cyclic GMP-AMP synthase (cGAS)/stimulator of interferon (IFN) genes (STING) and retinoic acid-inducible gene I (RIG-I)/mitochondrial antiviral-signalling protein (MAVS) pathways, respectively (21). These pathways activate complex downstream signalling *via* interferon regulatory factor 3 (IRF3)/TANK-binding kinase 1 (TBK1) and nuclear factor kappa B (NF-κB) that results in production of Type I IFN and other inflammatory cytokines (e.g. interleukin (IL)-1, tumour necrosis Factor (TNF)-α) (20).

Radiotherapy is a form of ionising radiation that hydrolyses water and forms reactive molecules, such as reactive oxygen species (ROS) and nitric oxide species (NOS), which can directly alter DNA, cellular components, and molecules in the extracellular matrix (ECM) (22). ROS and NOS can be derived both from these direct ionisation events or activated immune cells, and work with other DAMPs to accelerate lymphocyte and DC recruitment. These activated immune cells generate pro-inflammatory cytokines (e.g. TNF-α, IL-1β, IL-6, IL-12) (14, 23, 24), chemokines and growth factors leading to a sustained inflammatory response (22, 25).

#### 2.1.3 Immune cell recruitment and tumour-specific T-cell activation

Recent data suggest that radiation can enhance cancer cell antigenicity through upregulation of genes involved in DNA damage repair and cellular stress responses (20). Immune cell recruitment is subsequently increased *via* expression of adhesion molecules (e.g. intercellular adhesion molecule 1 (ICAM-1), vascular cell adhesion molecule 1 (VCAM-1) and E-selectin) (26) and chemokines (e.g. chemokine (C-X-C motif) ligand 16 (CXCL16)) (27). Within the appropriate inflammatory environment, DCs take up antigens in peripheral tissues and mature and migrate to draining lymph nodes, where they induce activation of naïve T-cells and differentiation into effector T-cells (28). Radiotherapy-induced ICD, as discussed above, increases tumour-associated antigen presentation that can lead to specific tumour-associated antigen T-cell priming, expansion of tumour reactive CD8+ T cells and infiltration into the tumour microenvironment (TME) (29). In summary, inflammatory DAMP signalling generates a favourable environment for activated DCs to process and cross-present tumour-derived antigens from irradiated cells as a “tumour vaccine”, to naïve T cells. These T cells subsequently can be primed and sustain a systemic tumour-specific immune response. The T-cell receptor (TCR) repertoire is also known to be shaped following radiotherapy, including when used in conjunction with immune checkpoint inhibitors (ICI) (30–32).

### 2.2 Immunosuppressive mechanisms triggered by radiotherapy

#### 2.2.1 Immunosuppressive cells within the tumour microenvironment

Whilst pro-inflammatory signalling can lead to a positive anti-tumour effect, cancer cells adapt to survive with mechanisms such as hypoxia resistance and unrestricted proliferation that can result in a state of chronic inflammation and evasion of immune surveillance (33–35). Evasion of immune recognition or immune escape (36) is now a recognised hallmark of cancer (9) and this inclination towards pro-tumour growth is mediated by changes in cytokine signalling (TNF-α, IL-1β, IL-6, IL-10 and TGF-β) (37, 38) and recruitment of TME-immunosuppressive immune cells such as tumour-associated macrophages (TAMs), myeloid-derived suppressor cells (MDSCs) (39) and regulatory T cells (Tregs) (40, 41).

PD-L1 (programmed death-ligand 1) expression is found to be elevated on tumour cells following irradiation due to interferon gamma (IFN-γ) release from tumour-infiltrating lymphocytes (TILs) (42) and TILs have increased expression of PD-1 (programmed death-1) following ex-vivo irradiation (43). A recent publication found that irradiation of colorectal cancer cells triggered an ATR-mediated DNA repair signalling pathway to upregulate CD47 and PD-L1, through engagement of signal-regulator protein α (SIRPα) and PD-1, respectively, to limit tumour-associated cross-presentation and suppression of innate immune activation (44).

Recruited MDSCs and TAMs can suppress T-cell function through antagonistic cytokine signals (45). Supporting data includes that from a phase I/II clinical trial testing the combination of radiotherapy and a primed DC vaccine in which non-responders had significantly higher baseline tumour levels of MDSCs (46).

Tregs are relatively more radioresistant than other lymphocyte subsets and radiotherapy may increase the infiltration by phenotypically and functionally suppressive Tregs within the TME (40, 41, 47). In several pre-clinical mouse models (B16/F10, RENCA and MC38), Tregs in irradiated tumours expressed higher levels of cytotoxic T-lymphocyte-associated antigen-4 (CTLA-4), 4-1BB (CD137, tumour necrosis factor receptor superfamily 9) and Helios compared with Tregs in non-irradiated tumours (47).

Cancer-associated fibroblasts (CAFs) can be the predominant component of the stroma in the TME and facilitate stroma-mediated radioprotection through multiple mechanisms. Following radiotherapy, CAFs can survive through formation of integrin-mediated attachments (48) and radioprotective integrin β-1 signalling (49). CAFs can promote an oxygen-rich, immunosuppressive and pro-inflammatory TME (50–52) resulting in increased tumour growth, invasion and metastasis (53).

Conversion of ATP to adenosine by CD39 and/or CD73 is a mechanism by which tumour cells can escape immune-surveillance by limiting the functionality of multiple potentially protective immune infiltrates, while enhancing the activity of immunosuppressive cell-types (54). CD39 and/or CD73 (over)expression has been found on the surface of tumour cells (55), CAFs (56) MDSCs (57), TAMs (58), Tregs and exhausted conventional CD4+ and CD8+ T cells (59–61).

#### 2.2.2 Tumour repopulation

One of the 4 “R”s of radiobiology is repopulation (5), and tumour repopulation during radiotherapy and chemotherapy is an important cause of treatment failure (62). Some tumours exhibit accelerated tumour repopulation following irradiation by paracrine caspase 3-dependent prostaglandin E_2_ (PGE2)-mediated signalling (63). Tumour repopulation may also be driven by a small number of cancer stem cells (CSC) which promote tumour growth following an insult, such as radiotherapy (64). Rapid proliferation of cancer cells is generally accepted as a prerequisite for most conventional chemotherapies and radiotherapy to be effective, and any senescent and/or quiescent tumour cells, such as CSCs, may be treatment-resistant (64). The CSC response to therapy may underpin why macroscopic tumour response to (chemo)radiation is not a robust predictor for clinical outcome, since small numbers of these relatively resistant and less immunogenic CSCs may survive to repopulate the tumour (64). However, *in vitro* pre-clinical data from human breast cancer cell lines (MCF-7 and T47D) have shown that radiotherapy can recruit CSC cells from a quiescent state into the cell cycle (65) and a CSC-druggable target in combination with radiotherapy would be useful.

As we have seen, radiotherapy can trigger key events leading to potent anti-tumour immune responses *via* production of immunostimulatory cytokines, DC recruitment, and T-cell recruitment and activation. However, these are negatively balanced by the potential for concurrent triggering of immunosuppressive cells within the TME and accelerated tumour cell repopulation. Targeting the DNA-damage response pathway (DDR) is an attractive approach to tip the scales towards maintaining positive immune anti-tumour states, which can be characterised as ‘pro-immunogenic’ and ‘pro-inflammatory’.

## 3 Targeting the DNA-damage response pathway

**Figure 1:**
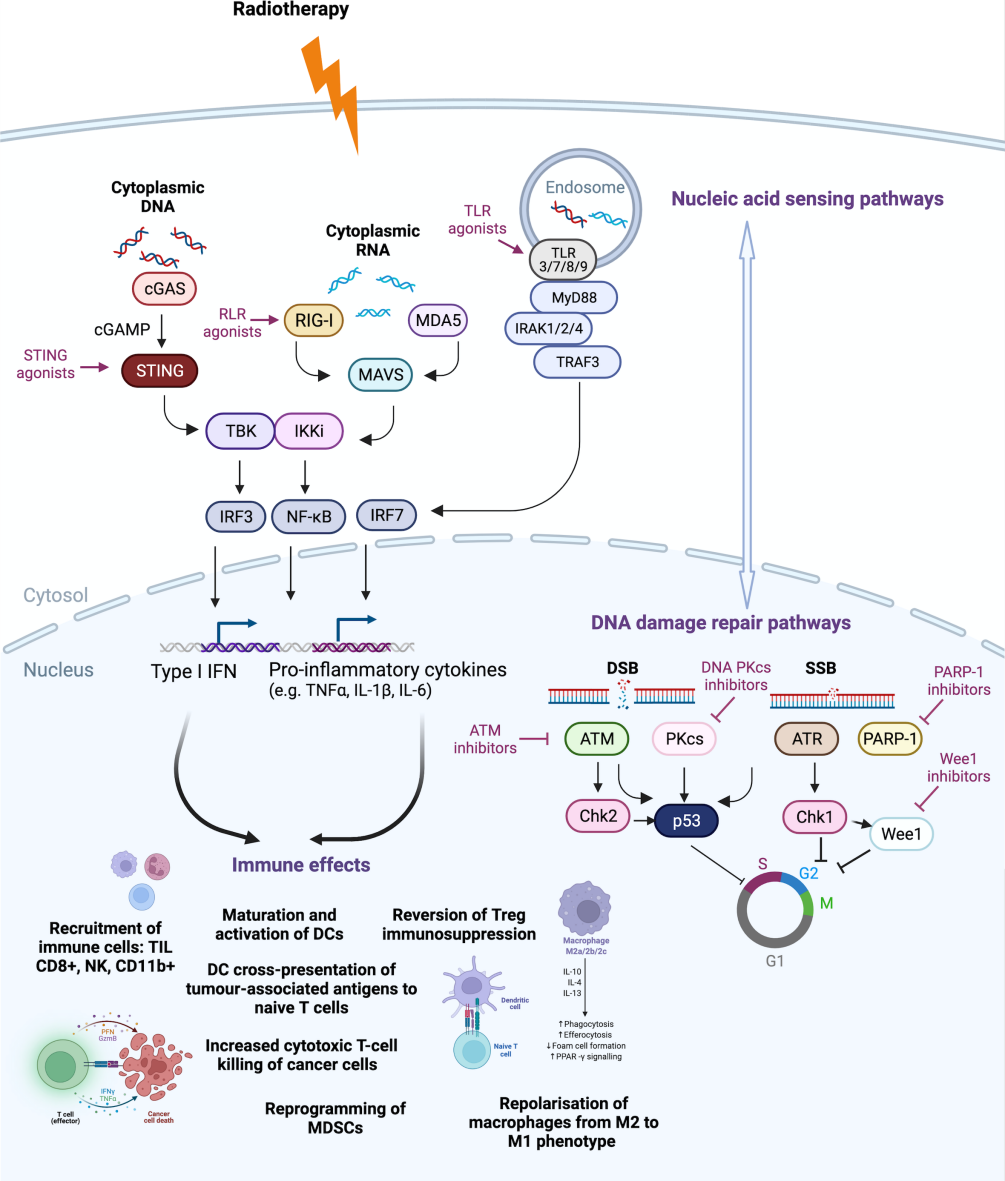
Druggable targets of the DNA damage response (DDR) pathway currently tested in clinical trials. Radiotherapy induces DNA damage and cell death. Nucleic acid sensing pathways detect cytoplasmic DNA and RNA to stimulate downstream pathways. Cytoplasmic DNA activates the Cyclic GMP–AMP synthase (cGAS) to produce cyclic GMP–AMP (cGAMP) that activates the stimulator of interferon genes (STING) pathway, leading to type I interferon (IFN) production. Radiotherapy-induced type I interferon (IFN) can induce RNA sensor activation through RNA polymerase III conversion of DNA to double-stranded RNA (dsRNA), radiotherapy-induced small non-coding RNA (sncRNA) or STAT1-induced dsRNA synthesis from endogenous retroviral elements (ERVs). These activate (RIG-I)-like receptors (RLRs), melanoma differentiation-associated protein 5 (MDA5) and retinoic acid-inducible gene-I (RIG-I), which also drives pro-inflammatory signalling through type I IFN and pro-inflammatory cytokine production. Toll-like receptors (TLRs) can recognise damage-associated molecular patterns (DAMPs) of single-stranded RNA (ssRNA), dsRNA or unmethylated CpG DNA in intracellular compartments such as endosomes, to lead to activation of nuclear factor-κB (NF-κB), mitogen-activated protein kinase (MAPKs) and interferon regulatory factors (IRFs). DNA damage repair mechanisms of single- (SSB) and double-strand breaks (DSB) are often upregulated by cancer cells to avoid cell cycle arrest or death. Inhibitors of DNA damage repair components, such as ataxia telangiectasia- mutated (ATM), ataxia telangiectasia and Rad3-related protein (ATR), DNA-dependent protein kinase, catalytic subunit (DNA-PKcs), poly(ADP- ribose) polymerase 1 (PARP-1) and Wee1 (Wee1-like protein kinase) function to propel the cell through the cell cycle, despite the presence of unrepaired damage, leading to accumulation of cytosolic DNA. This leads to cross-talk with the nucleic acid sensing pathway *via* activation of the cGAS-STING pathway and dsRNA stress pathway *via* promotion of ERV expression. These two pathways, through positive and negative cross-talk, shape the radiotherapy-induced DDR response that feeds into anti-tumour immune effects, including recruitment of tumour-infiltrating CD8+ T-cells, natural killer (NK) cells and CD11b+ innate immune cells, such as macrophages and neutrophils. Maturation and activation of dendritic cells (DCs) is increased, including DC cross-presentation of tumour-associated antigens to naive T-cells, which can become activated leading to T-cell-mediated cytotoxic-killing of cancer cells. Furthermore, the immunosuppressive effects of myeloid-derived suppressor cells (MDSCs) and regulatory T-cells (Tregs) can be reversed and macrophages can be repolarised from M2 to an M1 pro-inflammatory phenotype. Chk, checkpoint kinase; IKKi, inducible IκB kinase; IL, interleukin; IRAK, Interleukin 1 Receptor-Associated Kinase; MAVS, mitochondrial anti-viral-signalling protein; MyD88, Myeloid differentiation primary response 88; TBK, TANK-binding kinase 1; TNFα, tumour necrosis factor alpha; TRAF3, TNF Receptor-Associated Factor 3. Created with BioRender.com.

Radiotherapy causes cell damage, stress and death through induction of DNA lesions in the form of crosslinking, single-strand breaks (SSBs) and, most significantly, double-strand breaks (DSBs) (66). These processes induce a plethora of intracellular signalling pathways involved in detecting and repairing DNA damage. Targeting both DNA damage repair and DDR’s downstream cytosolic nucleic acid sensing pathways with small molecules in combination with radiotherapy can lead to increased immune activation and anti-tumour efficacy of these treatments (**Figure 1**).

### 3.1 DNA damage repair pathways

Radiotherapy induces double-strand breaks (DSBs) in cancer cell DNA, which results in genomic instability, cell cycle arrest, apoptosis or death *via* mitotic catastrophe (66). In response to radiotherapy, cancer cells can respond to exploit individualised DNA damage repair mechanisms for survival (67). Three primary DNA repair pathways have evolved to process DSB repair and maintain genomic integrity: homologous recombination, non-homologous end-joining (NHEJ) and alternative end-joining (68). Upregulation of these pathways is a mechanism by which cancer cells may acquire radioresistance and, accordingly, radiosensitisation strategies which inhibit radiation-induced DNA damage repair are expected to provide increased cancer control (66). When DNA repair is inhibited in cancer cells, this leads to accumulation of DNA damage, cellular stress and cell death which subsequently increases the likelihood of these cells triggering innate immune pathways and being recognised by anti-tumour immune surveillance.

#### 3.1.1 ATM and ATR inhibitors

ATM and ATR are both key mediators of the DSB signalling response that induce cell cycle arrest to facilitate DNA repair (69). In addition, conditions that activate ATM and ATR as part of DDR may also participate in regulating the innate immune system and alert it to potentially ‘dangerous’ tumour cells (70).

In response to DSB, the MRE11-RAD50-Nibrin (NBS1) (MRN) complex assembles at DSB sites to act as a DNA damage sensor that activates and recruits ATM to DSB sites (71). Briefly, when a cell triggers the DDR, ATM initiates a massive signalling cascade with the phosphorylation of hundreds of substrates, including p53 and checkpoint kinase 2 (Chk2). Activated p53 transactivates the expression of p21^Cip1/kip1^, which inhibits Cyclin Dependent Kinase (CDK) 2 and CDK4/6 to induce G1/S arrest (66). Chk2 in turn phosphorylates and inactivates Cell Division Cycle 25 (CDC25C), maintaining the inhibitory phosphorylation of CDK1 by Wee1-like protein kinase (Wee1) and Myelin Transcription Factor 1 (Myt1) to induce G2/M cell cycle arrest or apoptosis (66, 72). Inhibition of the ATM/Chk2 axis can lead to replication stress and accumulation of cytosolic DNA that subsequently activates the cGAS-STING-mediated innate immune response (73).

ATM was recognised as the defective gene in the inheritable human disorder, ataxia-telangiectasia (A-T) (74), and these patients have characteristic features including genomic instability and profound radiosensitivity (75). Deficiency of ATM-mediated signalling reactions causes sensitisation of cells to radiation (76), which has sparked interest in ATM as a therapeutic target for cancer treatment (69). Inhibition of ATM and ATR have the potential to improve radiotherapy outcomes as they are both key mediators of the DDR (69). Indeed, ATM inhibitors such as caffeine (77), wortmannin (78), CP-466722 (79), KU-55933 (80), KU-60019 (81) and KU-59403 (82) increase cell radiosensitivity (83, 84), particularly in p53 low/deficient and phosphatidylinositol 3-kinase (PI3K) highly-expressing cells (77, 85). In a preclinical study *in vivo* with KU60019 and radiotherapy, combination treatment enhanced TBK1 activity, type I IFN production, antigen presentation and increased CD8+ TILs; moreover, complete responders had established immunological memory (86) (**Table 1**). The ATM inhibitor (AZD1390) and radiotherapy is being investigated in a phase I clinical trial in brain cancer (NCT03423628). A dual ATM and DNA-PKc inhibitor (XRD-0394) and radiotherapy phase I trial is also recruiting (NCT05002140) (**Table 2**).

**Table 1:** Preclinical RT and DDR combination studies.

Target (drug), route	Additional therapy	Radiotherapy (RT)	Murine tumour model	Immunological effects	References
**DNA repair inhibitors**					
ATR inhibitor (AZD6738, ceralasertib), PO	**-**	2 Gy x 2	CT26 (colorectal cancer)	Combination treatment increased TIL CD8+ T cell infiltration, decreased TIL Treg cells, and promoted immunological memory. AZD6738 blocked radiation-induced PD-L1 upregulation to reduce number of TIL Tregs.	(87)
ATR inhibitor (AZD6738, ceralasertib), PO	**-**	2 Gy x 4	TC-1 (HPV- transformed lung epithelial cells)	Combination treatment showed enhanced type I and type II IFN signature, increased PD- L1 expression, increased numbers of DCs, T cells and NK cells.	(88)
ATR inhibitor (AZD6738, ceralasertib), PO	Anti-PD-L1	18 Gy in 3 fractions on days 1, 3, and 5	Hepa 1–6 cells (a C57/L murine liver cancer cell line) and H22 cells	AZD6738 further increased RT-stimulated CD8+ T cell infiltration and activation and reverted the immunosuppressive effect of radiation on the number of Tregs in mice xenografts. Triple combination with anti-PD-L1 boosted the infiltration, cell proliferation, enhanced IFN-γ production ability of TIL CD8+ T cells, decreased trend in number of TIL Tregs and exhausted T cells in mice xenografts. Triple therapy led to more long-lasting immunity with tumour rechallenge rejection.	(89)
ATR inhibitor (AZD6738, ceralasertib), PO	Anti-TIGIT, Anti-PD-1	20 Gy in four 5 Gy fractions per day (MOC2); 24 Gy in three 8 Gy fractions per day over 5 days (SCC7)	MOC2 and SCC7 HPV-negative murine oral squamous cell carcinoma cell lines	ATRi enhanced radiotherapy-induced inflammation in the TME with NK cells playing a central role in maximizing treatment efficacy. Anti-tumour activity of NK cells can be further boosted with ICI targeting TIGIT and PD-1.	(90)
ATM inhibitor (KU60019), PO	Anti-PD- L1	8 Gy single fraction	mT4 and KPC2 pancreatic cancer cell lines	Combination treatment further enhanced TBK1 activity, type 1 IFN production, and antigen presentation. ATM inhibition also increased PD-L1 expression, increased intratumoural CD8^+^ T cells and established immune memory.	(86)
DNA-PK inhibitor (M3814, peposertib), PO	Anti-PD-L1	5 Gy or 8 Gy single fraction	mT4 pancreatic cancer cell line	Radiation with DNA-PK inhibition increased cytosolic dsDNA and tumoural type 1 IFN signalling in a cGAS- and STING-independent, but an RNA POL III, RIG-I, and MAVS-dependent manner. Triple combination with anti-PD-L1 potentiated anti-tumour immunity with a significant increase in the number of CD4+ , CD8+ , and Granzyme B+ cells compared to radiation alone or radiation with M3814.	(91)
**Wee1 inhibitor**					
MK1775/AZD177, adavosertib, PO	Anti-PD-1	8 Gy single fraction	MOC-1 murine oral squamous cell carcinoma	Triple combination treatment efficacy is CD8-dependent. Radiation alone reduced neutrophilic myeloid-derived suppressor cells and increased Treg tumour accumulation, unchanged with the addition of AZD1775. T-cells from tumour-draining lymph nodes (TDLNs) from mice treated with the triple therapy demonstrated the greatest activation and IFNγ production upon exposure to MOC1 tumour antigen. Mice cured following triple agent treatment did not engraft tumours following rechallenge.	(92)
**STING agonists**					
Modified CDN derivative molecules, IT injection	**-**	10 Gy single fraction	Panc02 murine pancreatic adenocarcinoma cell line; SCC7 head and neck cancer model, MMTV-PyMT mammary carcinoma; 3LL lung adenocarcinoma model	Combination treatment showed early T-cell-independent and TNFα-dependent haemorrhagic necrosis, followed by later CD8+ T-cell-dependent control of residual disease.	(93)
**Toll-like receptor agonists**					
Imiquimod, topical	Cyclophosphamide	8 Gy x 3 consecutive days	TSA mouse breast carcinoma	Increased tumour infiltration by CD11c+, CD4+ and CD8+ cells. Tumour control abolished by CD8+ depletion. Combination treatment led to abscopal effect, long-term tumour-free mice rejected rechallenge showing immunological memory.	(94)
Imiquimod, topical	**-**	Whole-body RT 2 Gy single fraction	B16-F10 and B16-F1 melanoma	Combination treatment led to enhanced cell death via autophagy. Autophagy accelerated via ROS-mediated MAPK and NF-κB signalling pathways. Combination increased number of CD8+ T cells and decreased numbers of Treg and MDSCs in the tumour lesions. Combination enhanced systemic anti-cancer immunity by increasing the abundance of T cell populations expressing IFN-γ and TNF-α.	(95)
TLR7 agonist (R848), IV	**-**	10 Gy single fraction	B-cell lymphoma line A20, the T-cell lymphoma line EL4, and its ovalbumin-expressing derivative EG7	Combination treatment led to the longstanding clearance of tumour in T- and B-cell lymphoma-bearing mice. Combination therapy led to the expansion of tumour antigen-specific CD8+ T. Mice that achieved long-term clearance of tumour were protected from subsequent tumour rechallenge.	(96)
TLR7 agonist (DSR-6434), IV	**-**	KHT and CT26 tumours received a single dose of 25 or 15 Gy, or 5 daily fractions of 2 Gy, respectively.	CT26 colorectal or KHT fibrosarcoma tumours	Combination led to induction of type 1 interferon and activation of T and B lymphocytes, NK and NKT cells. Combination treatment primed an anti-tumour CD8+ T cell response. Long-term surviving mice had significantly greater frequency of tumour antigen-specific CD8+ T cells.	(97)
TLR7-selective agonist (DSR-29133), IV	**-**	2 Gy x 5	Syngeneic models of renal cancer (Renca), metastatic osteosarcoma (LM8) and colorectal cancer (CT26)	Administration of DSR-29133 led to the induction of IFNα/γ, IP-10, TNFα, IL-1Ra and IL-12p70. Combined therapy resulted in curative responses in a high proportion of mice bearing established CT26 tumours which was dependent on the activity of CD8+ T-cells, but independent of CD4+ T-cells and NK/NKT cells. Long-term surviving mice treated with combination were protected from subsequent tumour rechallenge.	(98)
TLR7/8 agonist (3M-011 (854A)), IP injection	**-**	2 Gy x 5	CT26 (murine colorectal carcinoma cell line) or Panc-02 (murine pancreatic carcinoma cell line)	*In vivo* depletion identified NK and CD8 T cells as the cell populations mediating the cytotoxic effects of treatment, while *in vivo* depletion of CD11c+ dendritic cells (DC) in CD11c-diphtheria toxin receptor (DTR) transgenic mice revealed DC as the pivotal immune hub in this setting.	(99)
TLR9 agonist (CpG oligodeoxynucleotide 1826), SC peritumoural or IT injection	**-**	Single dose (unspecified) or fractionated RT delivered in 1-9 Gy fractions twice daily, separated by 6-7 hours for 5 consecutive days for total dose of 10-90 Gy	Murine immunogenic fibrosarcoma tumour	Mice cured of their tumours by combined CpG oligodeoxynucleotide 1826 plus radiotherapy were highly resistant to SC tumour take or development of tumour nodules in the lung from IV injected tumour cells when rechallenged with fibrosarcoma cells 100 to 120 days after the treatment, suggesting the development of a memory response. CpG oligodeoxynucleotide 1826 also increased the radioresponse of the non-immunogenic fibrosarcoma tumour by a factor of 1.41 and 1.73 when CpG oligodeoxynucleotide 1826 was given SC and IT, respectively.	(100)
TLR9 agonist (CpG oligodeoxynucleotide 1826), peritumoural injection	**-**	20 Gy single fraction	Immunogenic sarcoma (FSa)	The CpG ODN-induced enhancement of tumour radioresponse was diminished in tumour-bearing mice immunocompromised by sublethal whole-body radiation. Tumours treated with combination showed increased necrosis, heavy infiltration by host inflammatory cells (lymphocytes and granulocytes), and reduced tumour cell density.	(101)
TLR9 agonist (CpG oligodeoxynucleotides), peritumoural injection		30 Gy in 10 fractions of 3 Gy over 12 days, or a single dose (2, 6 or 10 Gy)	Rat glioma cell lines 9L and RG2	Combination treatment efficacy was lost in nude mice compared to immunocompetent mice, underlining the role of immune cells in anti-tumour effects. Tumour infiltration by immune cells and expression within tumours of the CpG receptor, TLR9, were not modified by irradiation.	(102)
TLR9 agonist CpG oligodeoxynucleotides, SC injection	**-**	20 Gy single fraction	Lewis lung carcinoma (3LL) cells	TLR9 agonist alone expanded and activated B cells and plasmacytoid dendritic cells in wild-type mice and natural killer DCs (NKDCs) in B cell-deficient (B*−/−*) tumour-bearing mice. Combined treatment led to a strong tumour-specific humoral immune response with deposition of mouse IgG auto-antibodies in tumour tissue in wild-type mice whereas the number of tumour-infiltrating NKDCs increased in B^−/−^ mice.	(103)
**(RIG-I)-like receptor agonist (RLR)**					
dsRNA mimic polyIC by polyethylenimine (PolyIC(PEI)), IT cytoplasmic delivery	Low-dose cyclophosphamide, TLR agonist (polyIC), decitabine	Diffusing alpha-emitting radiation therapy (DaRT) Intratumoural Ra-224-coated seeds	4T1 triple-negative breast tumours Squamous cell carcinoma (SCC) tumour model SQ2	Splenocytes from PolyIC(PEI) and DaRT-treated mice, adoptively transferred to naive mice in combination with 4T1 tumour cells, delayed tumour development compared to naïve splenocytes. Delay in tumour development on re-challenge was demonstrated.	(104)

IV, intravenous; SC, subcutaneous; IP, intraperitoneal; IT, intratumoural; PO, oral.

**Table 2:** Selected clinical trials investigating radiotherapy in combination with DDR inhibitor and/or other agents.

Target (drug) & route	Additional therapy	Radiotherapy	Phase	Patient population	n	Response	Toxicity	NCT ID
**DNA repair inhibitors**								
ATM kinase inhibitor (AZD1390)	N/A	35 Gy over 2 weeks; 30 Gy over two weeks; 60 Gy over 6 weeks	I	Brain cancer	120	Recruiting	Recruiting	NCT03423628
ATR inhibitor (AZD6738)	None	20 or 30 Gy	I	Solid tumours	46	Active, not recruiting	Active, not recruiting	NCT02223923
ATR kinase inhibitor (BAY1895344)	Pembrolizumab	SBRT 3 fractions with 2-3 days between fractions	I	Recurrent head and neck squamous cell carcinoma	37	Recruiting	Recruiting	NCT04576091
ATR inhibitor (M6620)	Cisplatin; capecitabine	Not specified	I	Oesophageal cancer and other solid cancers	65	Recruiting	Recruiting	NCT03641547
DNA- PK inhibitor (M3814)	Avelumab	Hypofractionated in 5 fractions	I/II	Advanced hepatobiliary malignancies	92	Not yet recruiting	Not yet recruiting	NCT04068194
DNA- PK inhibitor (M3814)	Cisplatin	3 Gy x 10; 2 Gy x 33-35	I	Locally advanced tumours	52	Preliminary efficacy: in-field response (n=16): one patient had pCR, 4 PR, 7 SD, and 3 have not yet been evaluated. One patient was not evaluable.	Dose-escalation results reported (n=16 patients enrolled). The most frequent AEs were fatigue in 12/16 and nausea 8/16. No patients discontinued due to DLTs. Four DLTs were reported: grade 3 mucositis lasting > 7 days in 3/16 and odynophagia in 1/16, all recovered without sequelae. One fatal suspected unexpected serious AE considered as radiation pneumonitis occurred.	NCT02516813
DNA- PK inhibitor (M3814)	Capecitabine	45–50 Gy in 25–28 fractions over 5 weeks	Ib/II	Rectal cancer	165	Recruiting	Recruiting	NCT03770689
DNA- PK inhibitor (M3814)	Avelumab	30 Gy in 10 fractions over 2 weeks	I	Various advanced solid tumours	24	Recruiting	Recruiting	NCT03724890
DNA- PK inhibitor (M3814)	Temozolomide	60 Gy in 30 fractions over 6 weeks	I	MGMT promoter unmethylated glioblastoma or gliosarcoma	29	Recruiting	Recruiting	NCT04555577
DNA- PK inhibitor (M3814)	N/A	Not specified	I	Advanced head and neck cancer	42	Recruiting	Recruiting	NCT04533750
DNA-PK inhibitor (XRD-0394)	N/A	20 Gy in 5 fractions over 1 week	I	Various advanced solid tumours	38	Recruiting	Recruiting	NCT05002140
Dual ATM and DNA-PK inhibitor (XRD-0394)	N/A	20 Gy in 5 fractions over 1 week	I	Metastatic, locally advanced, or recurrent cancer	38	Recruiting	Recruiting	NCT05002140
PARP inhibitor (olaparib)	Durvalumab; Tremelimumab	30 Gy in 10 fractions over 2 weeks	I/II	Extensive stage small cell lung cancer	54	Recruiting	Recruiting	NCT03923270
PARP inhibitor (olaparib)	N/A	Not specified	I	Triple-negative breast cancer	24	Awaiting report	2/24 (8.7%) patients experienced acute grade 3 dermatitis related to RT. Olaparib-related toxicity grade 3-4 haematological toxicity was lymphopenia in 11/24 (45.8%) patients.	NCT03109080
PARP inhibitor (olaparib)	N/A	Unspecified standard radiotherapy treatment 5 days per week for 6 weeks	II	Inflammatory breast cancer	300	Recruiting	Recruiting	NCT03598257
PARP inhibitor (olaparib)	Durvalumab; carboplatin; etoposide	Not specified consolidative thoracic radiotherapy	I/II	Extensive-stage small cell lung cancer	63	Recruiting	Recruiting	NCT04728230
PARP inhibitor (olaparib)	N/A	High-dose 70 Gy in 35 fractions; elective neck 54.25 Gy in 35 fractions	I	Head and neck cancer	12	Active, not recruiting	Active, not recruiting	NCT02229656
PARP inhibitor (olaparib)	Temozolomide	2 Gy per fraction given once daily five days per week over 6 weeks, for a total dose of 60 Gy	I/IIa	High-grade gliomas	79	Recruiting	Recruiting	NCT03212742
PARP inhibitor (niraparib)	N/A	Not specified	I	Triple-negative breast cancer	20	Recruiting	Recruiting	NCT03945721
PARP inhibitor (niraparib)	Dostarlimab	Not specified	II	Triple-negative breast cancer	32	Recruiting	Recruiting	NCT04837209
PARP inhibitor (veliparib)	Temozolomide	30 daily fractions of radiation therapy 5 days per week for 6-7 weeks	II	Newly diagnosed malignant glioma without H3 K27M or BRAFV600 mutations	115	Active, not recruiting	Active, not recruiting	NCT03581292
PARP inhibitor (Veliparib)	N/A	50 Gy to the chest wall and regional lymph nodes plus a 10-Gy boost	I	Inflammatory or loco-regionally recurrent breast cancer	30	15 disease control failures during the 3 years of follow-up. 13 died (all after recurrence)	5 dose-limiting AEs occurred: 4 moist desquamation, 1 neutropenia. Crude Grade 3 toxicity was 10% at year 1, 16.7% at year 2, and 46.7% at year 3. At year 3, 6 of 15 surviving patients had severe fibrosis in the treatment field.	NCT01477489
PARP inhibitor (Veliparib)	Capecitabine	50·4 Gy in 1.8 Gy fractions daily, 5 consecutive days per week for 5·5 weeks	!b	Locally advanced rectal cancer	32	Tumour downstaging at surgery was noted in 22 (71%) of 31 patients; nine (29%) of 31 patients achieved a pathological complete response.	Common AEs included nausea in 17 patients (53%), diarrhoea in 16 (50%), and fatigue in 16 (50%). Grade 3 diarrhoea in three (9%) of 32 patients; no Grade 4 events.	NCT01589419
**Wee 1 inhibitor**								
Adavosertib (AZD1775)	Cisplatin	IMRT 5 days a week, once daily, Monday to Friday, for 6 weeks	I	Head and neck cancer	9	Completed	Completed	NCT03028766
Adavosertib (AZD1775)	Cisplatin	45 Gy or greater	I	Cervical, upper vaginal and uterine Cancers	33	Active, not recruiting	Active, not recruiting	NCT03345784
Adavosertib (AZD1775)	Cisplatin	70 Gy at 2Gy per fraction, 35 fractions, Monday to Friday over 7 weeks	I	Intermediate/high risk squamous cell carcinoma of head and neck	12	Completed	Completed	NCT02585973
Adavosertib (AZD1775)	Gemcitabine	52.5Gy in 25 fractions (2.1Gy/fraction), using intensity-modulated radiation therapy (IMRT) after chemotherapy	I/II	Unresectable adenocarcinoma of the pancreas	34	Median overall survival for all patients was 21.7 months (90% CI, 16.7 to 24.8 months) which was substantially higher than prior results combining gemcitabine with radiation therapy.	8/34 patients (24%) experienced a dose-limiting toxicity, most commonly anorexia, nausea, or fatigue.	NCT02037230
**Toll-like receptor agonists**								
TLR9 agonist (SD-101) intratumoural	N/A	4 Gy in 2 fractions over 2 days	I/II	Untreated indolent lymphoma	29	26/29 (89.7%) patients had tumour reduction at treated site. 24 (82.8%) patients had tumour reduction at non-treated sites.	Grade 1-2 drug-related AEs reported by all patients. Most common treatment-related side effect was a flu-like systemic reaction. 8/29 patients (27.6%) had grade 3 drug-related AEs. No drug-related grade 4 or serious AEs.	NCT02266147
TLR9 agonist (SD-101) intratumoural	Anti-OX40 (BMS-986178)	Low-dose not specified over 2 fractions	I	Low-grade B cell non-Hodgkin lymphoma	15	Recruiting	Recruiting	NCT03410901
TLR9 agonist (SD-101) intratumoural	Epacadostat	24 Gy in 8 fractions, 20 Gy in 5 fractions, 4 Gy in 2 fractions	I/II	Advanced solid tumours	20	Early outcome reported for 7 patients refractory to prior therapy with anti-PD-L1 checkpoint inhibition. In these patients, disease control rate (DCR) and abscopal DCR was 86% (6/7) and 100% (7/7), response rate was 43% (3/7), and abscopal response rate was 29% (2/7) including 2 patients with long-term durable complete responses.	Awaiting report	NCT03322384
TLR9 agonist (SD-101) intratumoural	Pembrolizumab; leuprolide acetate; abiraterone Acetate; prednisone	35 Gy in 7 fractions	II	Oligometastatic prostate cancer	42	Recruiting	Recruiting	NCT03007732
TLR9 agonist (SD-101) intratumoural	Ibrutinib	Not specified	Ib/II	Lymphoma	30	Early outcome reported for 13 patients treated with a median follow-up of 7.7 months. 6 of 12 evaluable patients had achieved a partial response (50% ORR) and 3 had achieved >50% reduction in distal tumour burden. Eight of 12 patients (66.7%) had experienced at least a 30% reduction in distal tumour burden.	AEs were consistent with known effects of ibrutinib and of CpG with no unexpected AEs to suggest synergistic toxicity. There were no grade 4 or 5 events. AEs led to ibrutinib dose reduction or discontinuation in 3 patients.	NCT02927964
TLR9 agonist (SD-101) intratumoural	Nivolumab	6-10 Gy per fraction to the injected lesion given on days 1, 3, 5, 8, and 10	I	Metastatic pancreatic adenocarcinoma	6	Active, not recruiting	Active, not recruiting	NCT04050085
CMP-001 intratumoural	Nivolumab; ipilimumab	Radiosurgery	I	Colorectal cancer metastatic to liver	19	Recruiting	Recruiting	NCT03507699
SD-101 intratumoural	Ipilimumab	Low-dose radiation therapy to 1 site of disease	I/II	Recurrent low-grade B-cell lymphoma	9	Completed	Completed	NCT02254772
Imiquimod (topical)	Cyclophosphamide	30 Gy in 5 fractions	I/II	Metastatic breast cancer	31	Completed	Completed	NCT01421017
Poly(ICLC) intratumoural	rhuFlt3L/CDX-301	2 Gy x 2	I/II	Lymphoma	11	Partial or complete response of treated tumour in 8/11 (72.7%). Six (54.5%) had stable disease/minor regressions at non-treated sites and three (27.3%) showed significant distant disease regression.	All AEs Grade 1 apart from 1 patient with G2 fever	NCT01976585
CpG- enriched TLR9 agonist (PF-3512676) intratumoural		4 Gy in 2 fractions over 2 days	I/II	Mycosis fungoides	15	One (6.7%) patient with complete clinical response, distant site clinical response seen in 5 patients (33.3%).	Mild injection site reaction and mild flu- like symptoms	NCT00185965

AEs, Adverse effects; DLTs, Dose-limiting toxicities; NCT, National Clinical Trial; N/A, Not Applicable.

ATR is activated by single-stranded DNA (ssDNA) structures that may arise at resected DNA DSBs or stalled replication forks. ATR is recruited *via* interaction of ATR-interacting protein (ATRIP) with ssDNA-bound replication protein A (RPA) (105). RPA-ssDNA complexes stimulate loading of the RAD9–HUS1–RAD1 (9–1–1) heterotrimer, that recruits DNA topoisomerase II binding protein 1 (TopBP1) which activates ATR (106). Once ATR is activated, downstream targets, including checkpoint kinase 1 (Chk1), promote DNA repair (107, 108), restart of stalled replication forks (109) and intra-S and G2/M cell cycle arrest (110, 111). In response to DNA damage, activation of the intra-S-phase cell cycle checkpoint slows progression of DNA replication to allow time for resolution (110, 111). In addition, the ATR-dependent G2/M cell cycle checkpoint is activated through degradation of cell division cycle 25A (Cdc25A) (111), and phosphorylation of Cdc25C phosphatase inhibits its ability to activate nuclear cell division cycle 2 (Cdc2) and, hence, mitosis entry (112). Most cancer cells are defective in DNA damage-induced checkpoints through e.g. p53 pathway mutations, which leads to dependence on the intra-S-phase and G2/M checkpoints for cell survival (69). Therefore, ATR inhibition will lead to accumulation of DNA damage, premature entry into mitosis, mitotic catastrophe and cell death (69).

ATR inhibitors include schisandrin B (113), NU6027 (114), NVP-BEZ235 (115), VE-821 (116), VE-822 (117), AZ20 (118) and ceralasertib (AZD6738) (119, 120). NVP-BEZ235 has been reported to induce marked radiosensitivity in Ras-overexpressing cancers (121), and NU6027 has been shown to increase sensitivity to DNA-damaging agents in breast and ovarian cell lines (114). VE-822 results in selective sensitisation of pancreatic tumours to radiation *in vivo* by increasing persistent DNA damage, decreasing cell cycle checkpoint maintenance and reducing homologous recombination repair (117). *In vitro*, ATR inhibition downregulates radiotherapy-induced programmed death-ligand 1/2 (PD-L1/2) expression to sensitise cancer cells to T-cell killing, in addition to potentiating DNA damage (122). Promising preclinical *in vivo* studies (**Table 1**) of the ATR inhibitor ceralasertib (AZD6738) in combination with radiotherapy have shown an enhanced type I/II interferon response and increased immune cell infiltrate (88), increased RT-stimulated CD8+ T cell infiltration (87, 89), NK-mediated anti-tumour immunity (90), as well as reversal of the Treg immunosuppressive effect (87, 89). In addition, further addition of ICI (i.e. anti-PD-1, anti-PD-L1, anti-TIGIT (T-cell immunoglobulin and ITIM domain)) to the ceralasertib (AZD6738) and radiotherapy combination further improved response and long-lasting immunity in a CD8+ (87, 89) and NK-dependent manner (90).

There are, to date, three early phase clinical studies investigating ATR inhibition and radiotherapy. PATRIOT, a phase I study of ceralasertib (AZD6738) in combination with palliative radiotherapy, has completed recruitment and is awaiting report (NCT02223923). BAY1895344 in combination with radiotherapy and pembrolizumab in recurrent head and neck squamous cell carcinoma (HNSCC) (NCT04576091) and M6620 with radiotherapy and chemotherapy in solid cancers (NCT03641547) are ongoing studies (**Table 2**).

A downstream target of ATR, Chk1, has also been investigated as a potential therapeutic target, due to its ability to activate intra-S and G2/M cell cycle checkpoints and modulate the replication stress response (123), particularly as a sensitiser to radiotherapy (124). Chk1 inhibitors, to date, include UCN-01 (125), LY2606368 (126), PF-00477736 (127), MK8776 (128) and CCT244747 (129), AZD7762 (130) and LY2603618 (131). Although there have been promising results in refractory acute myeloid leukaemia and advanced cancer with MK-8776 (132, 133) and LY2606368 (134), unfortunately severe adverse effects such as drug-related cardiac toxicity have also been reported during the clinical development of these drugs, e.g. AZD7762 (135). Thus far, no clinical trials are investigating the combination of Chk1 inhibition and radiotherapy.

#### 3.1.2 DNA-PKcs (DNA-dependent protein kinase, catalytic subunit) inhibitors

DNA-PK is pivotal for the initiation of DNA repair following DSBs, which ultimately results in recruitment of proteins involved in DNA damage repair progressing and ligating the broken DNA ends most recognised *via* the NHEJ pathway (136). Various cancer cell lines with reduced levels of DNA-PKcs show increased radiosensitivity compared to unirradiated controls (137–139) due to defective DNA DSB repair, inhibition of phosphorylated protein kinase B (Akt) on Ser473 and reduction of radiotherapy-induced transcription factor hypoxia-inducible factor-1 α levels (HIF-1 α) (138).

Given that DNA-PKcs is critical in radiotherapy-induced DDR, DNA-PKcs inhibition is an emerging therapeutic target for potentiating radiotherapy responses (140, 141), and many agents have already been tested in clinical trials. Non-selective DNA-PKcs inhibitors include wortmannin, which also inhibits ATM (142), and LY294002, which has a similar structure (143, 144). More selective DNA-PKcs inhibitors include NU7026 (145), NU7441 (146), IC86621, IC87102, IC87361 (147), vanillin (148), OK-1035 (149), SU11752 (150), BVAN08 (151), IC486241 (152) and NK314 (153). More recently, novel inhibitors have been discovered including M3814 (154), AZD7648 (155) and VX-984 (156). Doxycycline was first approved by US Food and Drug Administration (FDA) in 1967 as a broad-spectrum antibiotic and has recently been recognised to function also as an DNA-PK inhibitor (157). Mechanisms by which DNA-PKcs helps to sensitise to radiotherapy include prolongation of radiotherapy-induced G2/M phase arrest (158) and reduced repair of radiotherapy-induced DSB (147, 150, 159) leading to the induction of autophagic cell death and mitotic catastrophe (66).

In terms of DNA-PKcs inhibition leading to stimulation of the innate immune system, a recent study showed that combining radiation with M3814-induced DNA-PK inhibition increased cytosolic dsDNA and tumour type I interferon signalling in a cGAS-STING-independent, but RNA Polymerase III-, RIG-I- and MAVS-dependent manner, in pancreatic cancer models (91). Furthermore, radiotherapy and M3814 increased PD-L1 expression and sensitised to anti-PD-L1 treatment in poorly immunogenic pancreatic cancers (91). DNA-PKcs itself also functions as a DNA sensor that activates innate immunity. It has been reported to function as a PRR by binding to cytoplasmic DNA and can trigger a type I IFN response in a STING/IRF-3/TBK1-dependent manner (160) as well as a STING-independent manner *via* phosphorylation of heat shock protein HSPA8/heat shock cognate HSC70 (161). It is still unclear whether pharmacological inhibition of DNA-PKcs kinase activity may dampen anti-tumour immunity in contrast to inhibition of other DDR kinases described such as ATM or ATR.

Clinical studies of DNA repair inhibitors, M3814 (NCT04533750) and XRD-0394 (NCT05002140), in combination with radiotherapy are recruiting. In addition, triple combination of M3814 with radiotherapy and chemotherapy (NCT02516813, NCT03770689, NCT04555577) or anti-PD-L1 (NCT04068194, NCT03724890) are also awaiting report (**Table 2**).

#### 3.1.3 PARP inhibitors

PARP-1 has been the most extensively studied of the PARP superfamily and is a key regulator of DNA damage repair (162, 163). In response to DNA damage, such as that induced by radiotherapy, an initial response is poly(ADP-ribosyl)ation (PARylation) of proteins including nuclear DDR proteins, such as DNA-PKcs, to provide a local signal of DNA damage (163–165). Inhibitors of PARP generally function by inhibiting PARylation or suppressing PARP-1 release by ‘trapping’. PARP-1 inhibition has been reported to sensitise cancer cells to various forms of ionising radiation including conventional gamma irradiation (166, 167), proton-beam irradiation (167) and radionuclide therapy (168, 169) (**Table 2**). Although SSBs are primary repaired by PARP-1, its inhibition may not be lethal due to other available repair pathways, such as homologous recombination. However, deficiency in BRCA1/2 functionality, which are key components in the HR pathway of DSB repair, leads to synthetic lethality and selective sensitivity to PARP inhibition (170).

Beyond DNA repair, PARP-1 also plays an immunomodulatory role by regulating gene transcription of several immune cell types, modulating the stimulatory ability of DCs, and by directly affecting the differentiation and function of T and B cells (171, 172). PARP-1 knockout mice show reduced T helper type 2 (Th2) differentiation responses (172). PARP-1 is also involved in the differentiation of Foxp3+ regulatory T cells (Treg) and promotion of Treg cell apoptosis during inflammatory responses (172). PARP inhibitors generate cytoplasmic chromatin fragments with micronuclei characteristics which activate cGAS-STING, downstream type I interferon signalling and chemokine ligand 5 (CCL5) secretion in excision repair cross-complementation group 1 (ERCC1)-defective non-small cell lung cancer (NSCLC) cells (173). The capacity of PARP1 inhibitors to upregulate innate immune and inflammasome-like signalling events, such as cGAS-STING signalling, closely depends on their PARP1-trapping abilities (174, 175). In the context of viral infection, activated DNA-PK has been reported to phosphorylate PARP1 leading to its cytoplasmic translocation (176). Cytoplasmic PARP1 can then interact with and directly PARylate cGAS to inhibit its DNA-binding ability (176). This has implications to how PARP inhibition, in the context of cancer-induced genome instability, can positively modulate the host anti-tumour immune response.

Early PARP-1 inhibitors were non-specific and non-selective, such as nicotinamide (177), AG14361 (178) and 4-amino-1,8-naphthalimide (179). Newer PARP-1 inhibitors, such as olaparib and niraparib, are now used in routine clinical practice following approval by the FDA and European Union (180, 181). They are licensed for use in patients with advanced BRCA-mutated ovarian cancer, metastatic-castration-resistant prostate cancer with BRCA1/2 or ATM mutation (182), suspected germline HR repair gene mutated mCRPC who have progressed on enzalutamide or abiraterone (183) and, most recently, recurrent epithelial ovarian, fallopian tube or primary peritoneal cancer which has responded to first-line platinum chemotherapy (184, 185).

Combining PARP-1 inhibition and radiotherapy has been supported by preclinical studies. Particularly in BRCA1-mutant cancers, PARP inhibition showed radiation hypersensitivity in lymphoblastoid cells (186). In various models, PARP-1 inhibitors KJ-28d (187), ABT-888 (188) and the PARP-1/2 inhibitor MK-4827 (189) increased cancer cell radiation sensitivity.

Many clinical trials are underway investigating the combination of PARP inhibitors and radiotherapy, with addition of chemotherapy and/or immunotherapy agents (**Table 1**). The mechanisms underlying radiosensitisation by PARP inhibitors are still not completely clear and, indeed, recent studies have revealed a wider immunological role for PARP-1 that could potentially be exploited through new therapeutic approaches (190). For example, one study showed through multiomics profiling that macrophage-mediated immune suppression is a liability of PARP inhibition (191). Following this evidence, the rationale for combining CSF-1R blocking antibodies with PARP inhibitors led to reprogramming of the TME and significantly enhanced innate and adaptive anti-tumour immunity, which was CD8+-mediated in BRCA-deficient tumours *in vivo* (191).

#### 3.1.4 Wee1-like protein kinase (Wee1) inhibitors

Wee1 is a cell cycle checkpoint negative regulator at the G2/M transition. The process by which Wee1 activation leads to phosphorylation and inactivation of the cyclin B1/CDK1 complex blocking entry into mitosis is well described (192).

Emerging studies have highlighted the role of Wee1 directly and indirectly in immune signalling (193). For example, ineffective CDK-1-dependent nuclear laminin degradation abrogates apoptosis induction, leading to immune resistance in tumour cells (194). Accordingly, Wee1 inhibition reconstitutes CDK1 activity to reverse resistance of these cancer cells to immune attack (194). In various cancer models, Wee1 inhibition promotes accumulation of cytosolic dsDNA, leading to activation of the cGAS-STING pathway (**Figure 1**), increased type I interferon target gene expression when delivered alone (195), as well as in combination with ATR inhibitors (196) or immune checkpoint blockade (197). A STING-independent pathway by which Wee1 inhibition induces the interferon response has also been reported. In cGAS-STING-defective tumour models, Wee1 inhibition can upregulate immune signalling through the dsRNA anti-viral defence pathway by promoting expression of endogenous retroviral element (ERV) (198). ERVs trigger dsRNA stress and the interferon response, resulting in the recruitment of anti-tumour T-cells, and increased expression of PD-L1 with sensitisation to anti-PD-L1 blockade in multiple cancer models (198).

Wee1 inhibitors, some of which are concomitant CDK1 inhibitors, are promising as a combination partner with radiotherapy (199). This combination has shown synergistic effects in various cancer models (200–202). Wee1 inhibitors such as 681641 (203), PD0166285 (204) and adavosertib (MK1775/AZD1775) (92, 202, 205) have been reported to increase the radiosensitivity of cancer cells. Cancer cells very frequently harbour G1 checkpoint deficiencies and Wee1 inhibitor-mediated prevention of DNA repair following radiotherapy may lead to premature entry into mitosis and, ultimately, cell death *via* mitotic catastrophe (206). Other mechanisms include blocking radiotherapy-induced DNA damage repair (204) by impairing DNA repair protein RAD51 homolog 1 (RAD51) focus formation (202) and suppression of Sirt1 (silent mating type information regulation 2 homolog 1). Sirt1 interacts with and deacetylates HR-repair machinery proteins including Nibrin (NBS1) and RAD51, thus, Wee1-induced Sirt1 suppression impairs HR-repair activity (207).

Several clinical trials are exploring the combination of Wee1 inhibition by adavosertib (MK1775/AZD1775) with radiotherapy and chemotherapy (NCT03028766, NCT03345784, NCT02585973, NCT02037230) (**Table 2**). The emerging immune-mediating effects of Wee1 inhibition provide a strong rationale for its combination with immune checkpoint inhibitors (198).

### 3.2 Cytosolic nucleic acid sensing pathways

The ability to detect cytosolic nucleic acids by PRRs, arising from pathogens or disruption of cellular functions from genotoxic stress such as DNA damage, is part of the protective cellular response against infection or injury. These mechanisms are an evolutionary product of anti-microbial responses and can trigger an inflammatory signalling cascade and subsequent activation of the innate immune system. Targeting these nucleic acid sensing mechanisms has the potential to further amplify the DDR-induced anti-tumour innate immunity in conjunction with radiotherapy.

#### 3.2.1 Direct DNA sensing

##### 3.2.1.1 STING agonists

Stimulator of interferon genes (STING) is an endoplasmic reticulum adaptor that senses self and foreign cytoplasmic DNA, *via* cyclic GMP–AMP synthase (cGAS), and is crucial for effective innate immune signalling (208). Cytosolic DNA induces synthesis of the cyclic dinucleotide (CDN) cyclic GMP–AMP (cGAMP) from ATP and GTP by a cyclase enzyme called cGAS. cGAMP directly binds to STING to cause its dimerization and activation (209, 210), leading to activation of both NF-κB and IRF3 transcription pathways to induce expression of type I interferon, recruitment of immune cells, promotion of DC maturation and antigen-specific immune priming (211).

The cGAS-STING pathway is essential for anti-tumour T cell responses (212). One proposed mechanism is that CD8α^+^ DCs engulf apoptotic or necrotic tumour cells, and tumour cell-derived DNA triggers STING signalling in DCs (212–214). The subsequent type I IFN production by these DCs facilitates antigen cross-presentation and T-cell priming independent of the TLR or RIG-I/MAVS pathways (212). Recent studies have also suggested that STING signalling in the TME can suppress the immunosuppressive activity of MDSCs (215, 216). STING signalling is critical for radiation-induced anti-tumour responses (214) and, thus, it is an attractive potential treatment combination with radiotherapy. Preclinical data have shown that consideration needs to be given to radiotherapy dose per fraction as doses above 12-18 Gy induce the DNA exonuclease Trex1, which degrades the cytosolic DNA required to stimulate an effective STING-dependent type I IFN response (217).

The first generation STING agonist, 5,6-Dimethylxanthenone-4-acetic Acid (DMXAA), was originally developed as a vascular-disrupting agent (218, 219) and its anti-tumour effect is based on vascular necrosis leading to tumour starvation and haemorrhagic necrosis (218, 220). DMXAA has previously been shown to synergise with radiotherapy in mouse models in a hypoxia-preferential manner (221). However, the TME was found to remain immunologically sterile and tumours eventually progressed with time without durable protective anti-tumour immunity (222, 223). High local STING concentrations can lead to rapid T-cell apoptosis (224) whereas low-dose administration can lead to ‘vascular normalisation’ and favourably transform the TME to allow use of effective combinatorial anti-tumour immunotherapy (225–227).

There are two categories of STING agonists in clinical development: synthetic cyclic dinucleotides (CDNs) or non-CDN small molecules (228). These drugs are generally administered intratumourally due to their poor stability and bioavailability. This caveat limits their use to accessible tumours and recent efforts have been focused on development of STING agonists for systemic delivery (intravenously (228), orally (229, 230) and even as an inhalable nanoparticulate (231)). In addition, novel STING antibody-drug conjugates show promising preclinical results (232). There have only been a handful of preclinical studies investigating novel STING agonists with radiotherapy *in vivo* (**Table 1**). In mouse models, STING agonists synergise with radiotherapy to control local and distant disease and mediate rejection of tumour rechallenge (93, 231) *via* early T-cell-independent and TNF-α-dependent haemorrhagic necrosis, followed by a later stage of CD8 T-cell-dependent control (93). A number of clinical trials have looked into combining STING agonists with ICI or conventional chemotherapy (233); however, at the time of this review no radiotherapy and STING agonist combination clinical trials are in progress.

#### 3.2.2 Crosstalk with RNA sensors

##### 3.2.2.1 Toll-like receptor agonists

Toll-like receptors (TLRs) are a form of PRR expressed on sentinel immune cells which activate innate defence systems by detecting PAMPs. Genotoxic stress and DNA damage are increasingly recognised to signal through TLRs and cause the upregulation of TLR expression (234) *via* p53 (235). TLR signalling leads to maturation of APCs such as DCs, which are key mediators of T-cell activation and subsequent adaptive immunity. There is growing preclinical evidence that TLR agonists in combination with radiotherapy may lead to enhanced anti-tumour immunity, particularly through the mechanism of enhanced DC-mediated T-cell priming following radiotherapy (236). This occurs at various stages of this pathway; for example, TLR activation enhances type I IFN-signalling in many immune cells, modulates chemokine expression to enhance DC migration to lymphoid tissues (237–239) and upregulates CD80 and CD86 co-stimulatory molecules on DCs, which bind to CD28 on naïve T-cells for antigen/MHC-complex mediated TCR stimulation (240). TLRs can also stimulate DC-mediated release of IL-6 to dampen Treg suppressive signalling (241).

Given these observations, TLR agonists are seen as an attractive combination partner with radiotherapy. There have been numerous preclinical studies (**Table 1**) and early phase clinical trials (**Table 2**) of different TLR agonists, particularly of TLR3, TLR7/8 and TLR9, in combination with radiotherapy.

TLR3 senses dsRNA as a PAMP and polyinosinic-polycytidylic acid or poly (I:C) is a synthetic mimic of dsRNA which can stimulate TLR3-signalling pathways and lead to type I-IFN-dependent (242, 243) DC antigen cross-priming *in vivo* (244, 245). Poly(I:C) also has several immunostimulatory effects, including maturation and activation of DCs (246–248), T-cell stimulation (249, 250), enhanced cytotoxicity of Natural Killer (NK) cells (251–253), reprogramming of MDSCs (254) and repolarisation of macrophage populations from an M2 (classically activated macrophages) to M1 (alternatively activated macrophages) phenotype (255) (**Figure 1**). Pre-clinical studies exploring TLR3 agonists with radiotherapy in a radioresistant mouse model of lung cancer showed that poly(I:C) enhanced radiotherapy anti-tumour effects (256). The results from initial clinical trials have been disappointing, likely due to the short half-life of poly(I:C) (257). To address this, a degradation-resistant derivative polyinosinic-polycytidylic acid, and poly-L-lysine or poly(ICLC) was developed that has shown efficacy in clinical trials, although toxicity remains an issue (257). Preclinical studies in a murine lymphoma model have investigated the Fms-like tyrosine kinase 3 (Flt3)-ligand with radiotherapy and poly(ICLC) (258). Flt3-ligand is a cytokine which increases migration of DCs into the tumour and radiotherapy then stimulates maturation of DCs *via* ICD and HMGB-1 signalling for antigen uptake and processing (259). This combination with the addition of poly(ICLC) further maximises DC maturation and activation (246–248). There is a clinical study investigating intratumoral delivery of poly(ICLC) in combination with an in-situ vaccine rhuFlt3L/CDX-301 and radiotherapy which was well-tolerated and showed promising results (258) (NCT01976585) (**Table 2**). Two phase 2 studies in glioblastoma patients are also investigating the efficacy of poly(ICLC) in combination with radiotherapy (260, 261).

TLR7 and TLR8 detect guanosine or uridine-rich single-stranded RNA and their activation can directly induce MDSCs to lose their immunosuppressive function and acquire an APC-like phenotype that can induce tumour-specific T-cell responses (262), convert MDSCs to M1-like macrophages (263), activate NK cells (264–267) and revert Treg immunosuppressive effects (268). The imidazoquinolines are synthetic agonists for TLR7/8 of which topical imiquimod is the most extensively studied as well as being currently licensed for the treatment of superficial basal cell carcinoma (269). A preclinical study in breast cancer has investigated topical imiquimod in combination with radiotherapy and low-dose cyclophosphamide (94), and found that this triple combination had synergistic anti-cancer effects at both irradiated and unirradiated (abscopal) sites. Long-term surviving mice were able to reject tumour rechallenge, likely due to the establishment of anti-tumour immunological memory (94) (**Table 1**). A phase 2 clinical trial in metastatic breast cancer testing the efficacy of this triple therapy has finished recruiting (NCT01421017) (**Table 2**). Synergistic effects of subcutaneous TLR7 agonist and radiotherapy have also been observed in a preclinical model of melanoma (95) (**Table 1**). The efficacy of systemic delivery of the TLR7 agonists R848 (96), DSR-6434 (97), DSR-29133 (98) and 3M-011 (99), in combination with radiotherapy, has been explored in the treatment of several preclinical models of solid cancers. Dual therapy works synergistically to enhance tumour control, generate tumour-antigen-specific T-cells, suppress tumour growth (96–99) after rechallenge in long-term surviving mice (97) (98) and reduce the formation of distant metastases (99). Systemically-administered TLR7/8 agonists are not currently being investigated in a clinical setting; notably a phase I clinical trial investigating systemic TLR7 agonist ANA975 in chronic hepatitis C virus (270) had to be withdrawn due to excessive toxicity in extended preclinical studies (271), highlighting the need for caution when delivering systemic TLR7/8 agonists, especially in combination with radiotherapy (236).

Finally, TLR9 is expressed on APCs and B-cells and senses unmethylated CpG oligonucleotides present in bacterial and viral DNA (272–274). Again, TLR9 agonism can lead to activation and maturation of DCs, cytokine release from T helper type 1 (Th1) cells, differentiation of MDSC towards an M1 phenotype (275–279) and inhibition of Treg immunosuppressive effects (280). Several preclinical studies (281–284) have shown that TLR9 agonists can lead to anti-tumour effects in an NK- and CD8 T-cell-dependent manner (285). Preclinical studies showed enhanced tumour control in combination with radiotherapy in a model of murine fibrosarcoma and lung cancer (100–103), and induction of immunological memory by mice rejecting tumour rechallenge (102). The synergistic effects of radiotherapy and TLR9 agonists are dependent on a competent host immune system (102). Early clinical studies, although in small patient numbers, have tested TRL9 agonists in combination with radiotherapy. CpG-enriched oligodeoxynucleotide delivered intratumorally in combination with radiotherapy, 4 Gy in two fractions, led to overall objective response rates of 27% in the non-treated lesions of patients with relapsed low-grade B cell lymphoma (286).

##### 3.2.2.2 (RIG-I)-like receptor (RLR) agonists

RIG-I and melanoma differentiation-associated gene 5 (MDA5) are collectively (RIG-I)-like receptors (RLR) which detect cytosolic RNA and are a key PRR in anti-viral responses (287). RIG-I preferentially binds to short (>10 bp) dsRNAs whereas MDA5 detects long accessible dsRNAs (>2 kbp) (288, 289), and downstream signalling of either activates IRF3 and NF-κB pathways to induce type I IFN and other inflammatory cytokines. In the context of DNA damage, RIG-I interacts with X-ray repair cross complementing 4 (XRCC4) to impede formation of the XRCC4/LIG4 (DNA ligase 4)/XLF (XRCC4-like factor) at DSBs. High expression of RIG-I compromises DNA repair and sensitises cancer cells to irradiation treatment. In contrast, depletion of RIG-I renders cells resistant to irradiation *in vitro* and *in vivo* (290).

In the anti-tumour response, there is increasing evidence that RLR activation in various cancer models by RNA ligands can induce cancer cell apoptosis in a type I IFN-dependent (291), or -independent manner (292, 293). RIG-I signalling can induce ICD of ovarian and pancreatic cancer cells *in vivo* by systemic activation of DCs, NK cells and CD8+ T cells (294, 295). In a pancreatic cancer model, tumour-derived type I IFN activates DCs and CD8α^+^ DCs engulf apoptotic tumour material and cross-present tumour-associated antigen to naïve CD8+ T cells (296). RIG-I may also inhibit tumour growth indirectly through regulation of tumour hypoxia (297) and the gut microbiota (298). The efficacy of anti-cancer treatments such as radiotherapy and many chemotherapy agents has also been shown to depend on the RLR pathway through endogenous non-coding RNAs, and depletion of RIG-I in human tumours confers treatment resistance (299).

Harnessing the RLR-pathway through RLR agonists is an attractive therapeutic target and several RLR mimetics or agonists have been developed which have shown promise in preclinical studies. For example, a unique RIG-I agonist in the form of RNA stem-loop of 14 bp (SLR14), when delivered intratumorally, significantly inhibited B16 tumour growth locally and systemically in bilateral and tumour metastasis models, with cured mice developing immunological memory (300). SLR14 was mainly taken up by CD11b+ myeloid cells in the TME leading to subsequent increase in the number of CD8+ T lymphocytes, NK cells, and CD11b+ cells in SLR14-treated tumours (300). MK4621 (or RGT100), a synthetic RNA oligonucleotide RIG-I activator is currently in phase 1 clinical trials for the treatment of advanced/metastatic solid tumours (NCT03739138).

Combining RLR agonists and radiotherapy is an attractive strategy to activate multiple DDR pathways *via* cytosolic RNA sensing and radiotherapy-induced cytosolic DNA/DNA damage detection. *In vitro*, an RLR agonist Poly(I:C)-HMW (High Molecular Weight)/LyoVec™ [Poly(I:C)-HMW] sensitised *in vitro* human lung cancer cells to Fas ligand (FasL)-induced apoptosis by radiotherapy (301). *In vivo* intratumoral cytoplasmic delivery of the dsRNA mimic poly(I:C) by polyethylenimine (PEI), prior to diffusing alpha-emitting radiation therapy (DaRT), resulted in synergistic tumour and metastatic disease control. Furthermore, immunological memory was demonstrated, whereby splenocytes from treated mice adoptively transferred to naïve tumour-bearing mice, resulted in delayed tumour development and protection from rechallenge (104). Combining RLR-agonists and radiotherapy has not yet been translated into clinical practice and to the best of our knowledge there are no clinical trials investigating this combination.

## 4 Discussion

We have discussed in detail the various druggable targets related to the DDR pathway, in particular agonists of the nucleic acid sensing pathways and inhibitors of DNA damage repair mechanisms. Next, this review will explore the clinical challenges and implications of combining radiotherapy with DDR-targeted agents.

### 4.1 The role of conventional chemotherapy

Conventional chemotherapy has historically been used in the backbone of radical chemoradiation (CRT) in many locally advanced tumours such as rectal, cervical and head and neck cancers. Chemotherapy agents traditionally used as radiosensitisers include platin salts (e.g. Cisplatin, Carboplatin) or fluoropyrimidines (e.g. 5-fluorouracil or its prodrug Capecitabine), which trigger cell death by instigating DNA damage (302). Chemotherapy-induced cell death can lead to DNA leakage into the cytosol and trigger intrinsic STING pathway stimulation and activation of the immune system (303). Some may argue that investigating novel DDR-pathway specific agents is redundant given that chemotherapy may exert its anti-cancer effects partly by stimulating the innate immune system (303). However, it is recognised that chemotherapy (304), radiotherapy (305) or concomitant CRT (306) in various cancers can result in lymphocyte depletion which can potentially negate a sustained effective anti-tumour response. Lymphocyte depletion post-treatment is a poor prognostic factor in patients who have undergone radiotherapy for Stage III lung cancer (305) or CRT for newly diagnosed glioblastoma (306). Furthermore, defects in DDR signalling may contribute to chemoresistance in some cancer types (303) and, as such, development of specific DDR-targeting agents remains an important avenue for research.

### 4.2 Maintaining anti-tumour immunity using ICIs

The anti-tumour innate immunity initiated by radiotherapy and DDR inhibitors is likely to be complementary to the effect of immune checkpoint inhibitors (ICIs), which can sustain and maintain the adaptive arm of the anti-tumour immune response. For example, preclinical studies in lymphoma have shown that treatment with Flt3L, radiation and poly(ICLC) led to PD-L1 upregulation in both tumour cells and intratumoural DCs, and that the further addition of anti-PD-1 antibody led to improved local and systemic tumour control (258). There is an increasing number of early phase clinical studies investigating the addition of ICI with radiotherapy and DDR-targeted agents, such as TLR agonists (NCT03007732, NCT04050085, NCT03507699, NCT02254772) and DNA-PK inhibitors (NCT04068194, NCT03724890, NCT04576091, NCT03923270).

Clinical response to ICIs is typically predicted by tumour mutational burden and neoantigen load (307, 308). Preclinical data suggests that radiotherapy and DDR inhibitors may replicate the phenotype of high mutational and neoantigen burden and rationally direct therapeutic combinations with ICIs. However, the caveat is that radiotherapy-induced subclonal neoantigens may translate into poorer responses to ICI in some tumour types (307). The combination of radiotherapy and anti-CTLA-4 increases the diversity of TIL TCR repertoire, leading to increased tumour control *in vivo*; however, these tumours remain dominated by a small number of high-frequency T-cell clones (30, 32). It is still unknown whether it is more important to have an immune response against pre-existing tumour antigens or new radiotherapy-generated tumour antigens. As we await the results of the ongoing triple combination treatments (RT + DDR agents + ICI) in early phase clinical trials, further work is needed to investigate such combinations in the context of creation of subclonal neoantigens.

### 4.3 Tumour-specific radiosensitisation and the safety profile of combination therapy

A key principle of radiation oncology is that the dose delivered to the tumour is limited by the surrounding normal tissue organs-at-risk (OARs). Hence, strategies in designing clinical trials arguably should have some basis for a selective effect of any combination drug on the tumour (309). Preclinical studies in mouse models, for example, show that M3814, a DNA-PK inhibitor given with radiotherapy, shows marked improvement in tumour control (310). However, when translated into clinical practice, a clinical trial of M3814 with radiation (NCT02516813) reported enhanced normal tissue reactions including dysphagia, prolonged stomatitis and radiation dermatitis (311). Pre-clinical models are also severely limited in predicting long-term treatment toxicity in humans.

A further therapeutic challenge of using DDR pathway agents with radiotherapy is that there may be high variability in drug pharmacokinetics leading to varying degrees of radiosensitisation between tumour versus normal tissues, which makes it difficult to predict the therapeutic index for each individual patient (309). Therefore, unless there is a clear mechanism for tumour-specific radiosensitisation, clinical trials combining DNA repair inhibitors and radiotherapy may be severely compromised by unacceptable toxicity. Potential solutions may be an intratumoural route of drug delivery, as taken by certain trials of TLR9 agonists and STING agonists (**Table 2**), or conditional drug activation, such as with a hypoxia-activated DNA-PK inhibitor (312, 313). Increased knowledge of biomarkers and access to routine tumour profiling may guide the best selection of which DDR agent to use in a particular cancer subtype, for example PARP-inhibitors in BRCA-mutant or ATM/ATR inhibitors in p53-mutant tumours. Advances in radiotherapy delivery techniques using stereotactic techniques to irradiate tumour volumes highly selectively is a further way to reduce off-target combination effects of DDR-targeting agents. For example, a Phase I trial in recurrent head and neck squamous cell carcinoma investigating combining an ATR kinase inhibitor BAY1895344 with pembrolizumab and stereotactic body radiotherapy (SBRT) (NCT04576091) represents one such promising approach.

### 4.4 Radiotherapy planning, modality and scheduling with DDR delivery

In some occasions, radiotherapy can result in the regression of disease outside of the irradiated field in the so-called abscopal effect, which is thought to be immune-mediated (314). Inducing such systemic anti-tumour immune responses is likely highly dependent on radiotherapy dose and fractionation and these factors, therefore, need to be an important consideration in combination treatments with DDR agents and/or ICI (315).

Irradiation of regional lymph nodes in cancer treatment is common practice either with high doses in macroscopic disease or prophylactic lower doses, if lymph nodes are deemed to be at risk of harbouring micrometastatic disease. This approach has recently become more controversial given that we know these lymphoid organs have an important role in DC-mediated T-cell priming, activation and subsequent tumour infiltration following radiotherapy (31). Routine irradiation of regional lymph nodes may potentially deplete important immune cells and have a detrimental effect on the anti-tumour immune response (316).

The biological effects of radiotherapy, such as DNA damage complexity, depend on radiation quality and degree of linear energy transfer (LET). High LET radiation (e.g. protons, carbon ions, *α*-particle-emitting radionuclides) can differentially affect cell fate (317). For example, protons mainly induce apoptosis not necrosis which may reduce the leakage of nucleic acids into the cytoplasm to serve as danger signals, hence impacting on the innate immune response (317). The effects of radiotherapy were previously thought to be mainly due to nuclear DNA damage and their repair mechanisms. However, the outcome of irradiation depends also on the activation and regulation of other organelles that determine cellular metabolism, survival and immunological responses such as the mitochondria (318). Recent studies have shown that mitochondrial DNA DSBs activate a type I IFN response and mitochondrial RNA release into the cytoplasm triggers a RIG-I-MAVS-dependent immune response (319, 320). Low-dose versus high-dose radiation, as well as radiation quality, can also have different effects on mitochondria-mediated innate and adaptive immune responses (318). Interestingly, high LET particle radiotherapy which are more efficient in ROS production is reportedly more likely to lead to mitochondria-mediated apoptosis and anti-tumour immune responses (318, 321).

The most appropriate scheduling of DDR agents with respect to radiotherapy also needs to be investigated further. For example, a study investigating a novel TLR7/8 agonist in combination with radiotherapy showed that the optimal combination efficacy required the drug to be administered concurrently at the start rather than end of radiotherapy (98). However, another investigation of a TLR9 agonist showed maximum synergy was observed when mice received the agent three days after radiotherapy in the adjuvant setting (102). Clinical trials investigating TLR3 agonists used in the concurrent or adjuvant setting with respect to radiotherapy both showed activity (258, 260, 261, 322). More preclinical studies investigating the biological basis of optimal scheduling are required, although it may be that optimal scheduling may ultimately be both treatment- and tumour-specific.

## 5 Conclusion

Our increasing knowledge of the mechanisms of how radiotherapy-induced DDR interacts intimately with the host immune response is critical to the discovery of novel therapeutic targets and effective strategies against cancer. DDR-targeted agents are an exciting avenue for overcoming radioresistance and improving patient outcomes through enhancement of anti-tumour immunity. Understanding the molecular mechanisms and immunological effects of these DDR agents, through rigorous preclinical testing and translational analyses, is key to guiding rational clinical trial design in terms of drug route of delivery, schedules and choice of additional combination treatments, such as chemotherapy or immunotherapy.

## Author contributions

CC conceptualised this review and drafted the manuscript. AR, EP, MP, AM and KH contributed to the writing and critical revision of this article. All authors contributed to the article and approved the submitted version.

## Funding

This work was supported by the Wellcome Trust, ICR/RM NIHR Biomedical Research Centre, The Institute of Cancer Research/Royal Marsden Hospital Centre for Translational Immunotherapy, CRUK Head and Neck Programme Grant (C7224/A23275) and ICR/RM CRUK RadNet Centre of Excellence (C7224/A28724).

## Conflict of interest

The authors declare that the research was conducted in the absence of any commercial or financial relationships that could be construed as a potential conflict of interest.

## Publisher’s note

All claims expressed in this article are solely those of the authors and do not necessarily represent those of their affiliated organizations, or those of the publisher, the editors and the reviewers. Any product that may be evaluated in this article, or claim that may be made by its manufacturer, is not guaranteed or endorsed by the publisher.
